# Affinity Selection of MS2 VLPs as SARS-CoV-2 Vaccine Candidates Targeting Nucleocapsid Protein

**DOI:** 10.3390/v18070766

**Published:** 2026-07-13

**Authors:** Julianne Peabody, Chunyan Ye, Steven Bradfute, Bryce Chackerian, David S. Peabody

**Affiliations:** 1 Department of Molecular Genetics and Microbiology, University of New Mexico School of Medicine, Albuquerque, NM 87131, USA; bchackerian@salud.unm.edu (B.C.); dpeabody@salud.unm.edu (D.S.P.); 2 Department of Internal Medicine, University of New Mexico School of Medicine, Albuquerque, NM 87131, USA; cye@salud.unm.edu (C.Y.); sbradfute@salud.unm.edu (S.B.)

**Keywords:** COVID-19 vaccines, nucleocapsid protein, MS2 VLP, affinity-selection

## Abstract

Identifying antigens that elicit protective immunity is the key step for vaccine development. Here, we describe the use of the MS2 VLP platform to identify epitopes of SARS-CoV-2 structural proteins recognized by antibodies from COVID-19 patients, and to present those epitopes to the immune system as vaccines. We constructed an MS2 virus-like particle (VLP) library covering all four structural proteins of SARS-CoV-2 and affinity-selected vaccine candidates by biopanning on antibodies from infected humans. We focused on the structural proteins, reasoning that they are the most likely targets of a protective antibody response. The epitopes we found map almost entirely to the spike and nucleocapsid proteins. The VLPs displaying such epitopes were produced individually in *E. coli* and then tested for their potential as vaccines. While none of the affinity-selected spike-specific VLPs elicited neutralizing antibodies, VLPs displaying nucleocapsid epitopes induced protective immunity in a hamster model. This work illustrates the MS2 VLP platform’s capacity for the identification of new vaccine candidates and raises the possibility that VLPs displaying nucleocapsid epitopes could provide long-lasting protection against a range of virus variants.

## 1. Introduction

**Figure 1:**
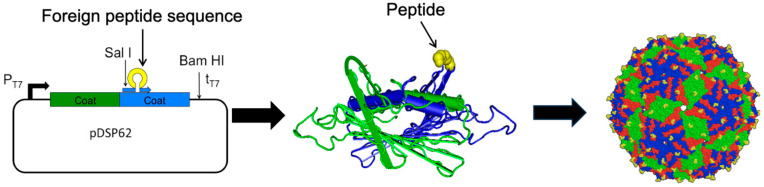
Peptides inserted into the downstream AB-loop of the MS2 coat protein single-chain dimer are displayed on the VLP surface. Encapsidation of the mRNA encoding coat protein and its guest peptide enables affinity selection as described in the text.

Identifying an appropriate antigen and presenting it to the immune system as an effective immunogen are key barriers to the development of a vaccine for any infectious agent. In diseases where natural infection confers immunity, it is often enough to use a killed or attenuated version of the pathogen itself. A more targeted approach might rely on immunogenic presentation of the subset of antigen epitopes normally responsible for a protective antibody response to natural virus infection. The MS2 VLP platform allows both the identification of antibody epitopes using a biopanning process akin to phage display, and their immunogenic display as vaccines. These capabilities depend on two features of the platform [1,2,3]. First, genetic insertion of peptide-encoding sequences into the AB-loop of the downstream copy of a specially engineered single-chain dimer version of the MS2 coat protein results in display of the peptides on the VLP surface (Figure 1). Because of their nanoparticulate and multivalent nature, VLPs elicit surprisingly long-lasting, high-titer antibodies to the peptides they display. Second, by naturally encapsidating its own mRNA, each MS2 VLP contains a copy of the genetic information for its own synthesis. Therefore, affinity-selected (i.e., biopanned) nucleic acid sequences can be recovered by reverse transcription and polymerase chain reaction after biopanning on antibody targets. These features of the MS2 VLP platform enable a process akin to phage display. Monoclonal antibodies can select epitopes or epitope mimics from random sequence peptide libraries, or, as in the present application, polyclonal antisera can select specific epitopes from antigen fragment libraries. DNA sequence analysis determines the identities of the selected peptides, and subsequent production of the VLPs in *E. coli* allows their utilization as vaccines. The MS2 VLP thus unites the epitope identification and immunogenic presentation functions in a single particle. By its nature, our approach focuses on antibodies and attempts to find epitopes able to elicit a protective humoral immune response. It makes no explicit attempt to address cellular immune responses.

Existing COVID-19 vaccines mimic an important aspect of natural infection by eliciting antibodies that bind spike protein and block viral entry. Their technical novelty and rapid development qualify these vaccines as some of the most notable achievements of modern biotechnology. Even so, they have shortcomings. The protection they confer against infection is relatively short-lived and is further compromised as viral variants arise that escape antibody neutralization. To counter these problems, occasional re-immunization with updated vaccines has become the norm. The value of these vaccines lies principally in their ability to protect against hospitalization and death. Antibodies against nucleocapsid protein are also a prominent component of the response to SARS-CoV-2 infection and are important contributors to natural immunity [4,5,6]. Because anti-N-antibodies cannot directly neutralize the virus, N-protein-based vaccines do not prevent infection, but they can significantly reduce its harmful effects.

Here, we describe the use of the MS2 VLP affinity-selection system to identify N-protein epitopes recognized by antibodies produced in humans during SARS-CoV-2 infection and show that these MS2-displayed epitopes can form the basis of a vaccine that elicits a protective antibody response. But why make an epitope-specific, VLP-based vaccine targeted to the N-protein rather than one based on the N-protein itself? Several other approaches to N-targeted vaccines immediately come to mind, and indeed some have already been described [7,8,9,10,11,12,13,14,15,16,17,18,19,20]. Most are based on delivery of the complete antigen, either in the form of purified protein or as an N-encoding nucleic acid delivered as mRNA, as DNA, or by any of a variety of viral vectors. Such vaccines normally elicit relatively short-lived antibodies, but a long-lived high-titer antibody response is essential for any vaccine whose effect depends on antibody presence at the time of infection. VLP-based vaccines are well known for their ability to elicit durable high-titer antibodies. In the case of an epitope-specific MS2 VLP-based Human Papillomavirus (HPV) vaccine, for example, the antibody titers it provoked decreased only slightly over the entire lifetimes of immunized mice. Furthermore, even a single dose of a vaccine based on VLPs of the HPV L1 protein provokes high-titer protective antibodies in humans that last for years [21,22,23].

## 2. Materials and Methods

### 2.1. Antigen Fragment Libraries and Deep Sequence-Coupled Biopanning

We constructed an antigen-fragment peptide library by inserting 9–14 amino acid peptides into the AB-loop of the MS2 coat protein single-chain dimer (Figure 1). In aggregate, they represent the complete sequences of all four SARS-CoV-2 structural proteins: spike, nucleocapsid, membrane, and envelope. The library was designed by computationally fragmenting each protein sequence into peptides of 9, 10, 11, 12, 13, and 14 amino acids, with each peptide overlapping its neighbor by three amino acids. In other words, taking the 9mer as an example, we scanned through each sequence with a 9 amino acid window in 3 amino acid increments. We repeated this process for each peptide length. We then reverse-translated the amino acid sequences of each of the roughly twelve thousand peptide sequences into their corresponding nucleotide sequences with *E. coli* codon preferences and then, using a microchip-based method, synthesized oligonucleotide primers with flanking sequences suitable for introduction of the foreign sequences into the MS2 coat protein’s AB-loop. We constructed the libraries in plasmid pDSP62 using methods described in previous studies [3,24,25]. In effect, the library is a composite mixture of six individual libraries that together represent the entire SARS-CoV-2 structural proteome in peptides of lengths from 9 to 14 amino acids displayed on the MS2 VLP surface.

We synthesized library VLPs in *E. coli*, purified them, and subjected them to affinity-selection as described [3,26]. We obtained plasma samples from patients with ongoing or recent SARS-CoV-2 infections [27] and purified IgG using protein G-coupled magnetic beads (Thermofisher 10004D, Waltham, MA, USA). We then incubated 500 µg IgG with 40 µg antigen fragment library VLPs and recovered VLP–antibody complexes by binding them to protein G-coupled magnetic beads. After extensive washing, we eluted them by brief incubation in 0.1 M glycine, pH 2.7, and then neutralized with 1 M tris, pH 9. The RNA contained in the VLPs was reverse transcribed and PCR amplified. During amplification, we labeled individual samples with barcoded primers and then subjected them to Ion Torrent sequencing. Using custom MATLAB scripts (version 2021a, The Mathworks, Natick, MA, USA), we mapped individual selected sequences onto the primary sequences of the four SARS-CoV-2 structural proteins. The enrichment of any given selected sequence relative to its abundance in the native VLP library identified specific antibody-bound epitopes. We inserted the sequences most often recognized into VLPs, which we then over-expressed in bacteria and purified for use in animal immunizations.

### 2.2. Animals

Two groups of five 4–6-week-old female Balb/c mice (Jackson Laboratories, Bar Harbor, ME, USA) were immunized intramuscularly in the hind limb. One group was immunized with 10 µg MS2 VLPs that bore no foreign peptide and was boosted 21 days later. The other received a pool of MS2 VLPs displaying nucleocapsid-derived epitopes totaling 10 µg (1.25 µg each VLP) and was similarly boosted at 21 days. Blood was collected monthly for eleven months via retro-orbital bleed under 3% isoflurane inhalation anesthetic using oxygen as a carrier.

Hamster experiments were conducted under light isoflurane anesthesia. Four groups of 2–3-month-old male Syrian hamsters (Charles River, Wilmington, MA, USA; 9 animals per group; determined by power analysis) received intramuscular injections in the hind limb. Group 1 received 10 µg recombinant nucleocapsid protein (Sino Biological 40588-V08B, Paoli, PA, USA) in 50% (*v*/*v*) CFA and was boosted 21 days later with 10 µg recombinant nucleocapsid protein in 50% (*v*/*v*) IFA. Group 2 received 10 µg wild type MS2 VLPs and was boosted 21 days later with same. Group 3 received 10 µg recombinant spike protein (Sino Biological 40589-V08H4, Paoli, PA, USA) in 50% (*v*/*v*) CFA and was boosted 21 days later with 10 µg recombinant spike protein in 50% (*v*/*v*) IFA. Group 4 received a pool of MS2 VLPs displaying nucleocapsid-derived epitopes (Table 2) totaling 10 µg (1.25 µg of each VLP) and was boosted 21 days later with the same. An additional group received no immunizations and served as a naïve control. Approximately 3 weeks after the final immunization, blood was collected via retro-orbital bleed for analysis by ELISA. Approximately 6 weeks after the final immunization, hamsters were challenged intranasally with 10^5^ pfu SARS-CoV-2 isolate USA-WA1/2020 (BEI Resources, NIAID). Three days post-infection, 3 of the 9 hamsters were sacrificed by CO_2_ asphyxiation and their lungs were removed to determine viral titers. The remaining 6 animals were weighed on each of the 21 days after infection. Analysis of weights (daily) and viral titers was performed by lab or animal facility personnel who were blinded to group identity.

Animals were divided into groups randomly and housed in the same location. No animals were excluded from analysis. No adverse events were observed in any animal studies following immunization.

### 2.3. Characterization of Antibody Responses

Blood was collected from mice or hamsters, and sera were separated from insoluble factors by centrifugation. End-point dilution titers were determined by ELISA using Immulon 2HB microtiter plates (Thermo Scientific 3455, Waltham, MA, USA). Volumes were 50 µL per well, incubation was at room temperature on an orbital rocker, and plates were thoroughly washed with PBS between each step. Plates were coated with recombinant spike or nucleocapsid protein (20 ng per well in PBS) and incubated overnight at 4 °C, and then blocked with 100 µL 0.5% (*w*/*v*) dry milk in PBS for 1–2 h. Sera were diluted in the blocking buffer and incubated on the plate for 2.5 h. HRP-conjugated goat anti-mouse or HRP-conjugated goat anti-Syrian hamster (Jackson Immunoresearch, West Grove, PA, USA) secondary antibodies were diluted in blocking buffer and then incubated in wells for 1 h. Color development was with 3,3′,5,5′-Tetramethylbenzidine (TMB, Millipore Sigma T0440, Sigma-Aldrich, St. Louis, MO, USA). Acidification with an equal volume of 1% HCl stopped the reaction within 10 min of developer addition. Readings (OD_450_) greater than twice the average background were considered signal positive.

### 2.4. Determining Viral Lung Titers

Three days post-infection, 3 of the 9 hamsters in each group were sacrificed by CO_2_ asphyxiation, and viral titers were determined as previously described [27]. Briefly, lungs were homogenized, cellular debris was removed by centrifugation, and dilutions of the supernatant were applied to Vero E6 cells and incubated at 37 °C for 2 h. After removal of the lung supernatant, viral overlay medium was added and the cells were incubated for 2 days at 37 °C, at which point the cells were fixed and stained with crystal violet.

## 3. Results

### 3.1. Affinity Selection of Antigen Fragment Libraries on Patient Sera

We constructed antigen-fragment libraries that display 9–14 amino acid peptides that together represent the entire SARS-CoV-2 structural proteome on the MS2 VLP surface. We biopanned the library on plasma samples collected from three convalescent donors (numbered 2, 4, and 9) and from nine acutely infected hospitalized individuals (1, 2, 3, 7, 8, 10, 11, 12, and 13) at time points defined from the day of study enrollment (day 0) in the study [27]. Table 1 characterizes the sera with respect to their neutralizing titers for SARS-CoV-2 (by PRNT) and their ELISA endpoint dilution titers for the N-protein. It is well known that neutralization generally reflects the binding of antibodies to a subset of epitopes on the spike protein. The presence of neutralizing antibodies is presumably irrelevant for our identification of N-protein epitopes but, along with the anti-spike and anti-N-protein ELISA titers, serves as a measure of the strength of the anti-SARS-CoV-2 response in these patients. Consistent with the known immunogenicity of nucleocapsid protein, anti-N-protein titers in our samples were typically high, with most individuals showing endpoint dilution titers between about 10,000 and more than 600,000. The considerable heterogeneity in the strength of the antibody responses is probably due to differences in individual immune system responses and likely also reflects differences in the time of blood collection relative to the start of infection. For most patients, plasma was collected on day 0 and again on day 7. The anti-N titer at the second collection was generally much higher than that at the first. It should also be noted that our three control sera from uninfected individuals showed some reaction with the N-protein, but the titers were substantially lower than nearly all the COVID-19 patient samples. We speculate this reflects the presence of cross-reacting antibodies elicited by prior infection with other coronaviruses. Our samples were all collected in the early days of the pandemic, so prior infection with SARS-CoV-2 seems unlikely.

### 3.2. Epitope Profiles

**Figure 2:**
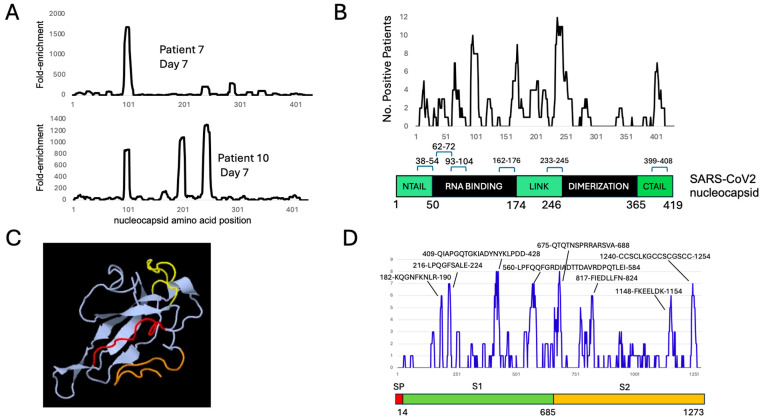
(**A**) The epitope profiles of two representative individuals. The *y*-axis shows the fold-enrichment plotted against each nucleocapsid amino acid by residue number on the *x*-axis. (**B**) The epitope positivity profile for the nucleocapsid protein. The *y*-axis shows the number of patients (out of 12) whose sera gave at least 100-fold enrichment of any given amino acid by residue number on the *x*-axis. The locations of the six major nucleocapsid epitope regions identified in the plot are shown on a schematic of the nucleocapsid protein sequence. (**C**) Experiments focused on six immunodominant N-protein epitopes, three of which reside in unstructured regions. The others are found in surface loops within the stably folded RNA-binding domain and are shown here with residues 62–72 in red, 93–104 in yellow, and 162–176 in orange. (**D**) The epitope positivity profile for the spike protein mapped onto a schematic of the spike sequence.

We performed biopanning with these samples and then carried out Ion Torrent sequence analysis on the resulting affinity-selected VLP populations. We determined the degree of enrichment of any given peptide by measuring its abundance in the selected population against its abundance in the starting library. In Figure 2A, we show typical nucleocapsid epitope profiles from two individuals. Profiles for all patients for both spike and nucleocapsid proteins are shown in Supplementary Figures S1 and S2, where the fold enrichment of every amino acid is plotted against its numbered position in the protein sequence. Peaks represent peptides enriched by antibody binding and therefore correspond to specific linear epitopes. Although the VLP libraries contain peptides representing all four virus structural proteins, nearly all the affinity-selected peptides map to the spike and nucleocapsid proteins, presumably reflecting the relative immunogenicities of individual epitopes in the four viral structural proteins. Only a few cases of peptides mapping to membrane or envelope proteins were found, and although we cannot exclude the possibility that these could function as vaccines, we concentrated our experiments on the immunodominant spike and nucleocapsid epitopes.

Individual patient epitope profiles for the spike protein (Figure S1) and for the N-protein (Figure S2) show considerable differences in both the number and the identities of epitopes that their sera recognize. Responses to the spike focused on only a few epitopes, and although some were shared by different patients, none was shared by all. Similarly, most individuals responded to several N-protein epitopes. In fact, more than half were positive for at least five epitopes, and although some were selected by multiple individuals, only one (the region between 233 and 252) was common to all twelve. Ten donors recognized peptide 92–104, nine recognized 162–171, and seven selected epitopes at 62–72 and 399–408. The response of one of the twelve individuals seemed to focus on a single peptide. To facilitate comparisons and to identify the most often recognized epitopes, we condensed the individual data into the summary plots of Figure 2B,D. We arbitrarily defined a patient to be “positive” for a given amino acid whenever affinity selection led to its enrichment by more than 100-fold. We then plotted the number of such positive patients against each amino acid position in the spike and nucleocapsid sequences. We focused subsequent experiments on those most frequently recognized.

### 3.3. Testing the Vaccine Potential of Affinity-Selected Epitopes

Because we were aware of the spike protein’s importance as a vaccine target from the beginning, we initially focused our attention on its epitopes (Figure 2D). To test their vaccine potential, we constructed plasmids to produce the relevant VLPs in *E. coli*, purified them [28], and then immunized mice. ELISAs of the resulting sera showed that they yielded sera with anti-spike end-point dilution titers between 10^2^ and 10^4^. Despite their ability to bind the spike, however, none showed any significant neutralizing activity in PRNT, even at the highest concentrations (20-fold dilution) we tested. In retrospect, this is unsurprising since we now know that the spike’s neutralizing epitopes are generally conformational in nature [29,30,31] and for that reason are not represented among the relatively small peptides displayed in our libraries.

**Figure 3:**
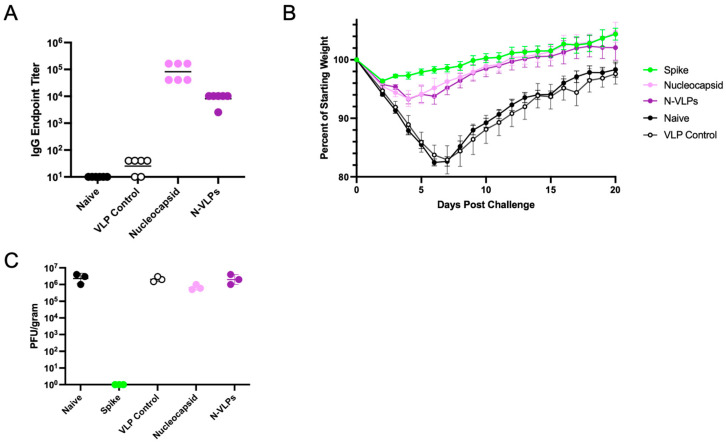
(**A**) End-point dilution titers of anti-nucleocapsid antibodies in sera from hamsters immunized with the indicated antigens. (**B**) Animal weights after infection with SARS-CoV-2 in hamsters immunized with spike protein, nucleocapsid protein, MS2 VLPs (VLP control), or MS2 VLPs displaying nucleocapsid epitopes. An unimmunized (naive) group is also included as a control. (**C**) SARS-CoV-2 titers in the lungs of hamsters three days after infection with SARS-CoV-2.

The nucleocapsid epitopes gave a more encouraging result. Eight different MS2 VLPs were constructed, each displaying one of the peptides listed in Table 2. Together, they represented the six distinct N-protein epitopes whose relationship to the N-protein structure is shown in Figure 2B,C. Antibodies to the N-protein are not expected to have neutralizing activity, so instead of using PRNT, we assessed the ability of the N-VLPs to prevent severe disease in a hamster model. When immunized with an equal mixture of N-VLPs (without adjuvants), hamsters typically generated antibody end point dilution titers around 10^4^. This is about an order of magnitude lower than titers elicited by full-length recombinant N-proteins (Figure 3A), a difference probably accounted for, in part, by the use of a strong adjuvant (Complete Freund’s Adjuvant) with the intact N-protein. In contrast, we used no adjuvant at all with the N-VLPs. The presumed presence of multiple epitopes in the intact protein probably also plays a role in producing higher titers to the full-size protein.

We infected the hamsters with 1 × 10^5^ pfu SARS-CoV-2 and followed the animals’ weights for 21 days. In hamsters, SARS-CoV-2 infection is seldom fatal but generally causes severe weight loss [32,33], which is considered to correspond to relative disease severity. Control animals, both the unimmunized and those vaccinated with unmodified MS2 VLPs, lost about 18% of their body weights by day 7, and then slowly returned to their starting weights by day 21. As a positive control, we also immunized with the intact spike protein. Spike is well known, of course, to have a protective effect, but even these animals lost about 5% of their body weight by day 2. They soon began to recover, however, and regained their starting weights by day 10. Animals immunized with the N-protein or with N-epitope-displaying VLPs were also significantly protected against severe weight loss (Figure 3B). N-VLP and nucleocapsid-immunized animals lost only 7% of their body weight by day 4, had already begun to recover on day 5, and returned to their starting weights by day 13, continuing to grow until the study’s end at day 21. Notably, immunization with N-VLPs protects as well as the N-protein itself, despite the difference in antibody titers.

We removed lungs on day 3 from three animals in each group and determined their virus titers by plaque assay. Control animals immunized with recombinant spike protein had no detectable virus in their lungs due to the ability to elicit virus-neutralizing antibodies. Animals immunized either with the N-protein or with N-VLPs showed no obvious inhibition of virus production at 3 days, even though they strongly inhibited weight loss (Figure 3C). Since immunized animals recover so much more rapidly than unimmunized or MS2-immunized controls, we think they probably also clear the virus more rapidly, but at three days, it may be too early to see much effect.

**Figure 4:**
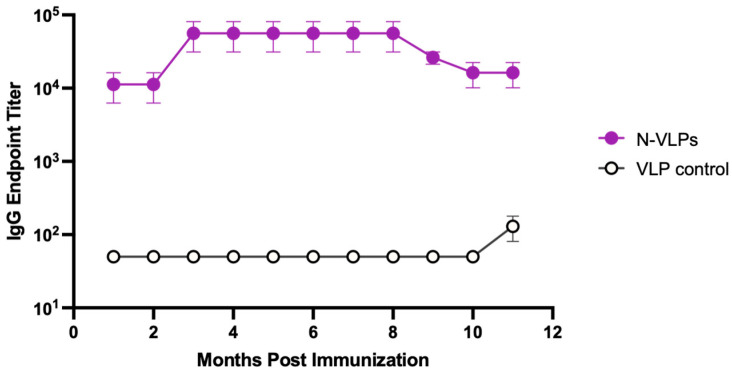
Longevity of anti-nucleocapsid antibody responses in mice.

To test the durability of responses to the N-VLPs, we immunized mice as described and performed ELISAs on sera collected monthly for 11 months (Figure 4). Anti-N-protein titers declined only a little over the course of the experiment, consistent with results observed previously with other MS2 VLP-displayed epitopes [23].

## 4. Discussion

The continuing threat of coronavirus emergence underscores the importance of research into alternative vaccine strategies, not just against new SARS-CoV-2 variants, but also for coronaviruses generally. In a perfect world, a vaccine would fully prevent infection. Like many other vaccines, existing COVID-19 vaccines, all of which target the spike protein, fall short of this goal. They are nevertheless considered efficacious because they prevent an infection’s severest consequences. Because antibody titers gradually decline, their main long-term value is not in protecting against infection per se, but in preventing death and hospitalization. Our results suggest the possibility that nucleocapsid-targeted vaccines could have a similar effect. Furthermore, the nucleocapsid’s amino acid sequence is more conserved, making us wonder whether it could elicit broader protection, perhaps obviating the need to update vaccines to counter constantly emerging spike variants. The nucleocapsid sequence is nearly identical across known SARS-CoV-2 variants and even shows significant conservation across different coronaviruses. In Table 2, we show the epitopes utilized in our studies and compare them to the same regions of several SARS-CoV-2 variants and of SARS-CoV.

With so much attention focused on the spike protein, it is easy to overlook the N-protein’s potential as a vaccine antigen, even though much of the normal human immune response is targeted to it. The N-protein is not restricted to the virion, of course, but is abundantly present on infected cell surfaces [34], which presumably accounts both for the N-protein’s immunogenicity and for its role as a target for antibody-mediated immunity. Indeed, the protection afforded by N-protein immunization should not surprise us, even though it presumably cannot be a target of virus-neutralizing antibodies, which normally function by inhibition of the interaction of spike protein with its cell-surface receptor. For one thing, we have known for more than 40 years that an N-protein-specific monoclonal antibody protects mice from mouse hepatitis coronavirus infection [35]. For another, we now recognize that adoptive transfer of anti-N antibodies can protect mice from the effects of infection by SARS-CoV-2 [36], and that anti-N immunoglobulin in COVID-19 patient sera is a component of the passive transfer of immunity to humans [4]. The mechanisms of this protection have not been fully defined, but antibody-dependent cellular cytotoxicity (ADCC) probably accounts for at least some of it [37]. Neutralization of the N-protein’s ability to suppress innate immunity by cytokine sequestration might also contribute [34]. In addition, antibody-N-protein complexes can enter cells where they interact with the Fc receptor of the TRIM21 ubiquitin ligase. This targets N for proteolysis, which in turn could promote the antigen cross-presentation that enhances a cytotoxic T-cell response [38]. It is also likely that interaction with TRIM21 inhibits virus growth simply by stimulating intracellular degradation of viral proteins [39]. Although we emphasize here the protective role of antibodies, we should bear in mind that the nucleocapsid can also be a target of cell-mediated immunity [40]. Our method is specifically intended to identify the targets of antibodies, and although we cannot yet rule out a role for cell-mediated immunity in our experiments, VLPs are best known for their ability to elicit a powerful antibody response. We therefore suspect the protection we observe is primarily antibody-mediated.

Our experiments illustrate both the power and limitations of the MS2 VLP platform for vaccine discovery. In the beginning, we hoped to find VLPs displaying spike epitopes that would elicit neutralizing responses. We now know that the relevant spike epitopes are conformational and therefore not amenable to a method that relies primarily on the display of relatively small peptides. This presumably explains why we were unable to identify any spike epitopes that elicited neutralizing antibodies, despite our method’s efficiency in finding them. Nucleocapsid protein, on the other hand, probably represents a richer source of linear epitopes, especially with its disordered linker and N- and C-terminal sequences. Indeed, three of our six epitopes (Figure 2 and Table 2) map to these disordered regions. The other three are found in surface loops of the stably folded RNA-binding domain. Since we tested the N-VLPs as a pool, we do not yet know whether all six epitopes contribute to immunity.

If the protective effect we observe depends on the presence of anti-N-protein antibodies at the time infection occurs, the longevity of protection should in turn rely on the long-term persistence of those antibodies. It is well known that VLPs, even when administered in a single dose in humans, can elicit antibodies that persist for years, perhaps even decades [21,22,41]. Meanwhile, monomeric antigens generally provoke shorter-lived responses. We showed previously in mice that antibodies to a Human Papillomavirus epitope displayed on MS2 VLPs persist at high titers for what amounts virtually to the animal’s lifetime [23]. Antibodies to the N-VLPs seem similarly long-lived, suggesting that the protection they confer should persist.

In addition to possible utility as a stand-alone vaccine, VLPs displaying nucleocapsid epitopes have the potential to expand and enhance the immunity provoked by existing SARS-CoV-2 vaccines. Furthermore, due to the nucleocapsid protein’s stronger sequence conservation, an appropriately designed nucleocapsid-based vaccine might expand protection to SARS-CoV-2 variants and perhaps even to other coronaviruses. Further, existing vaccines against SARS-CoV-2 largely employ mRNA or virus-vectored vaccines, which have several recognized disadvantages. Likely because they require that protein be synthesized by host cells, responses to these vaccines vary greatly between individuals, sometimes to such an extent that some people are considered non-responders [30,42,43,44,45]. Immunity following mRNA vaccination rapidly declines, and repeated vaccination with vectored vaccines may reduce efficacy [46,47]. Such limitations argue for further exploration of protein-based immunogens. A nucleocapsid-targeted vaccine, especially one that elicited a sufficiently long-lived antibody response, could provide an adjunct or even an alternative to existing vaccines.

## Figures and Tables

**Table 1:** Characteristics of the patient sera used in this study. Individuals denoted by simple numbers are hospitalized, infected patients. Those here called “donors” are convalescent.

Patient	Day After Admit	Anti-N ELISA Titer	Neut. Titer
1	0	163,840	640
	14	655,360	160
2	0	40,960	640
	7	10,240 *	160
3	0	10,240	0
	7	40,960	640
7	0	10,240	640
	7	163,840	2560
8	0	640	0
	7	40,960	160
10	0	163,840	0
	7	655,360	640
11	0	2560	0
	7	163,840	1280
12	0	640	0
	7	163,840	2560
13	0	10,240	40
	7	655,360	5120
Donor 2	NA	10,240	160
Donor 4	NA	10,240	0
Donor 9	NA	10,240	40
Negative control 1	NA	640	ND
Negative control 2	NA	640	ND
Negative control 3	NA	2560	ND

* Estimated by back calculation from the titer of purified IgG.

**Table 2:** VLPs used for immunization. Amino acid substitutions found in some important SARS-CoV-2 variants and in SARS-CoV are also shown.

VLP	Epitope Sequence	SARS-CoV-2 Variants	SARS-CoV
MP26	38-KQRRPQGLPNNTASWFT-54		
MP27	38-KQRRPQGLPNNT-49		
MP28	40-RRPQGLPNNTAS-51		
Nuc1	62-EDLKFPRGQGV-72	D63G (Delta)	D64E, K66R
MP30	93-RIRGGDGKMKDL-104	G99S(Omicron)	I94V, D103E
Nuc2	162-PQGTTLPKGF-171		
MP31	233-KMSGKGQQQQGQT-245	S235F (Alpha)	N234V
Nuc3	399-ADLDDFSKQLQ-408	K405E (Delta)	L401M, K407R

## Data Availability

The data presented in this study are available on request from the corresponding author.

## Supplementary Materials

The following supporting information can be downloaded at https://www.mdpi.com/article/10.3390/v18070766/s1, Figure S1. Spike protein epitope profiles for individual patient sera. The fold-enrichment (Y axis) of each amino acid in the affinity-selected VLP population relative to its abundance in the unselected VLP library is plotted against its position in the protein sequence (X axis). Figure S2. Nucleocapsid epitope profiles for individual patient sera. The fold-enrichment (Y axis) of each amino acid in the affinity-selected VLP population relative to its abundance in the unselected VLP library is plotted against its position in the protein sequence (X axis).

## Abbreviations

The following abbreviations are used:

VLP	virus-like particle
MS2 VLP	virus-like particle of bacteriophage MS2
N-VLP	MS2 VLPs bearing nucleocapsid epitopes.
CFA	complete Freund’s adjuvant
IFA	Incomplete Freund’s adjuvant
ELISA	enzyme-linked immunosorbent assay
pfu	plaque forming unit
PRNT	plaque reduction neutralization test
