# Supplementary material for: Affinity Selection of MS2 VLPs as SARS-CoV-2 Vaccine Candidates Targeting Nucleocapsid Protein
Source: Viruses. 2026 Jul 13;18(7):766. doi: 10.3390/v18070766 (PMC13431509; doi:10.3390/v18070766)
Supplement: Supplementary file 1 [file viruses-18-00766-s001.zip › viruses-4382800-supplementary.pdf]

Supplementary Information

Figure S1.
Spike protein epitope profiles for individual patient sera. The fold-enrichment (Y axis) of each amino acid in the affinity-selected VLP population relative to its abundance in the unselected VLP library is plotted against its position in the protein sequence (X axis).

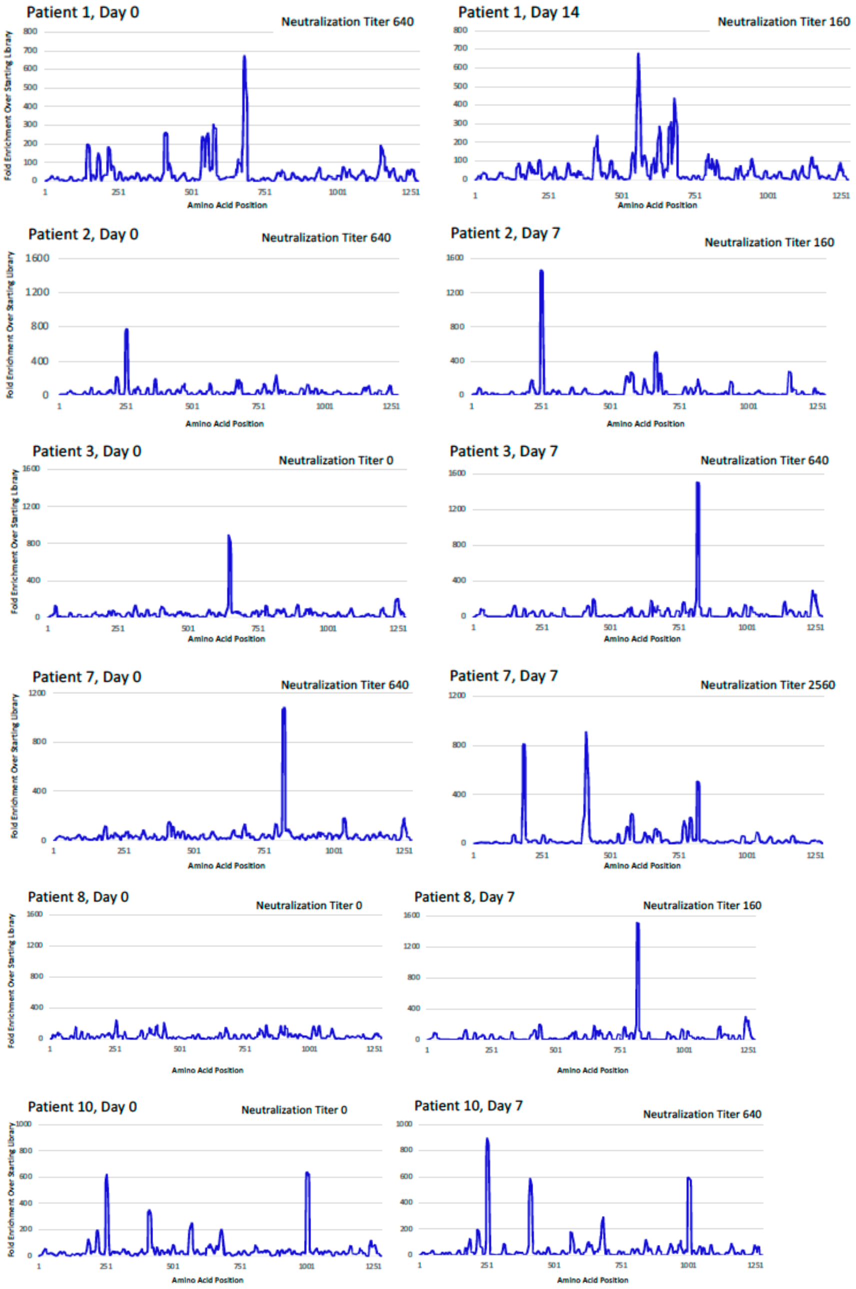

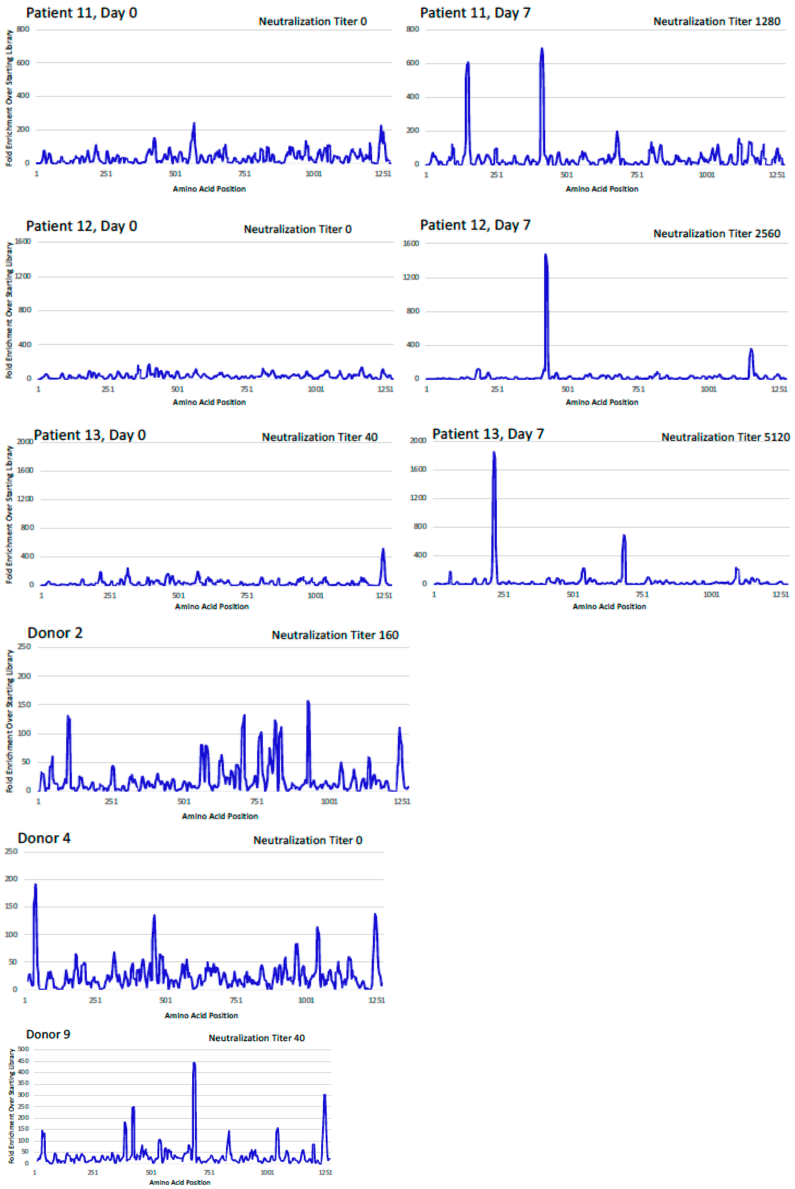

Patient Summary Data

Figure S2

Nucleocapsid epitope profiles for individual patient sera. The fold-enrichment (Y axis) of each amino acid in the affinity-selected VLP population relative to its abundance in the unselected VLP library is plotted against its position in the protein sequence (X axis).

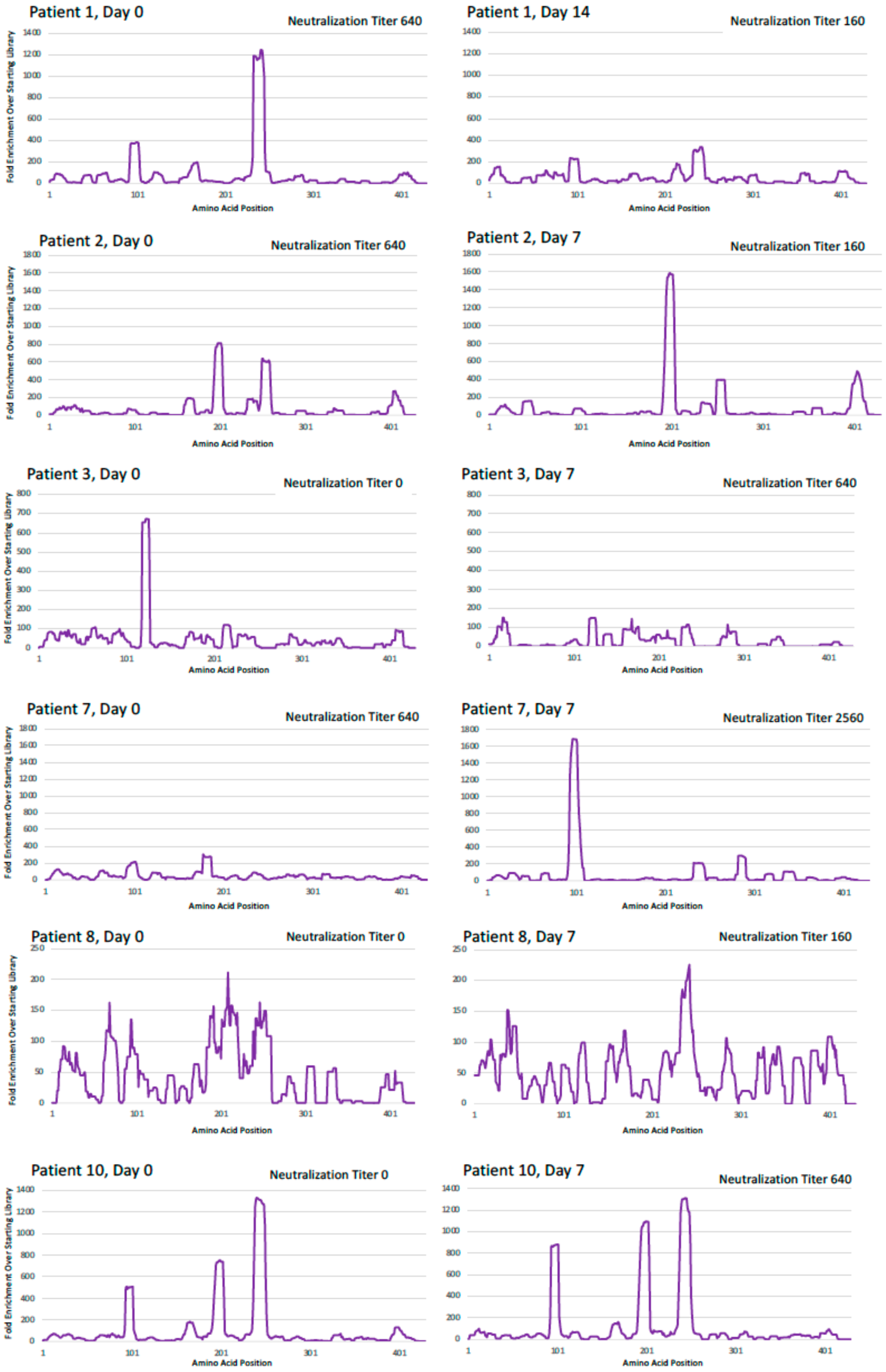

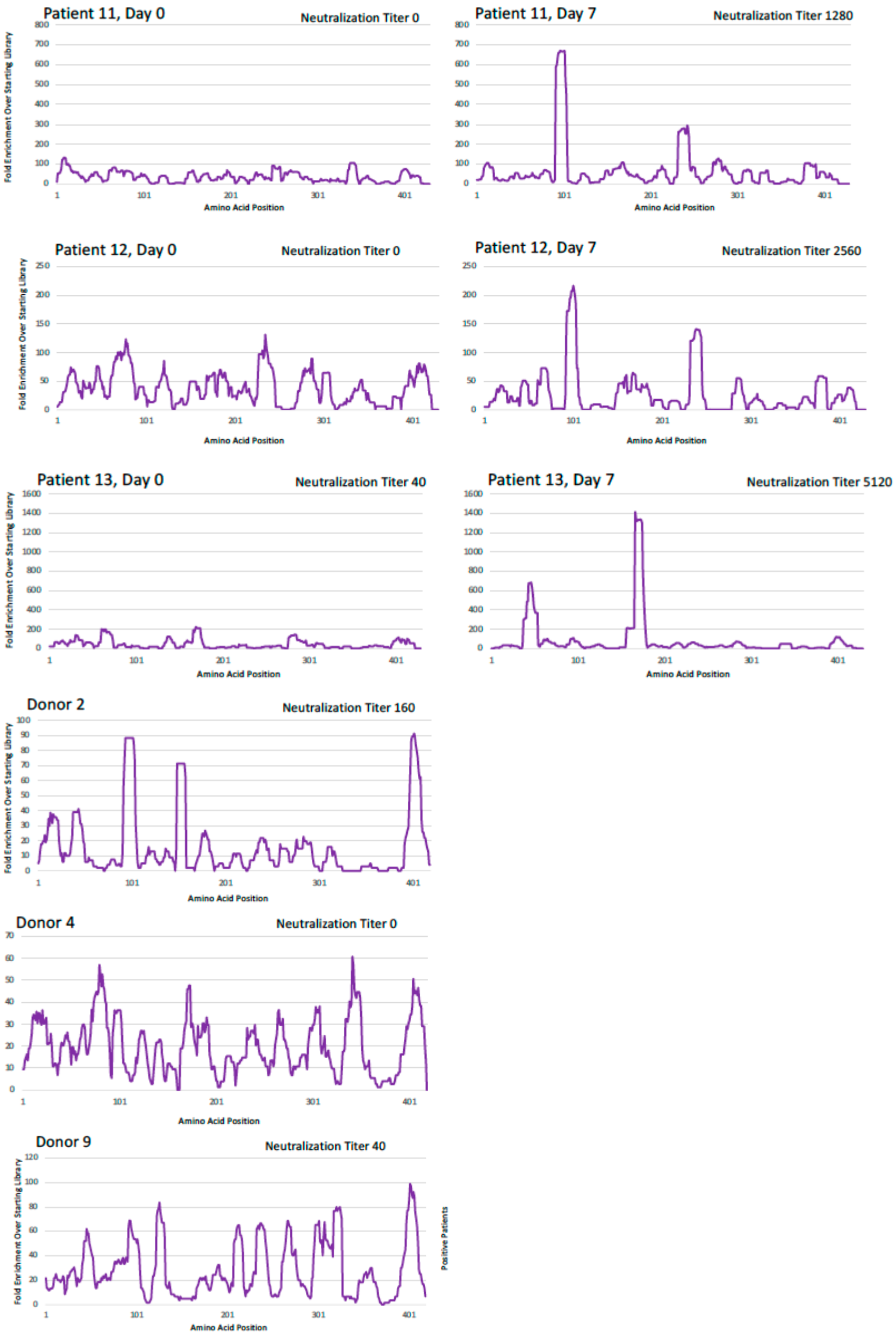
Patient 11, Day 0
Neutralization Titer 0
Patient 11, Day 7
Neutralization Titer 1280
Patient 12, Day 0
Neutralization Titer 0
Patient 12, Day 7
Neutralization Titer 2560
Patient 13, Day 0
Neutralization Titer 40
Patient 13, Day 7
Neutralization Titer 5120
Donor 2
Neutralization Titer 160
Donor 4
Neutralization Titer 0
Donor 9
Neutralization Titer 40
Fold Enrichment Over Starting Library
Amino Acid Position
Positive Patients
